# Regulation of Cerebral Blood Flow

**DOI:** 10.1155/2011/823525

**Published:** 2011-07-25

**Authors:** Eric C. Peterson, Zhengfeng Wang, Gavin Britz

**Affiliations:** ^1^Department of Neurological Surgery, University of Washington, Seattle, WA 98195, USA; ^2^Division of Neurosurgery, Duke University, Durham, NC 27710, USA; ^3^Department of Neurosurgery, Hospital of Zhengzhou University, Zhengzhou 450001, Henan Province, China

## Abstract

The control of cerebral blood flow is complex, and only beginning to be elucidated. Studies have identified three key regulatory paradigms. The first is cerebral pressure autoregulation, which maintains a constant flow in the face of changing cerebral perfusion pressure. Flow-metabolism coupling refers to the brains ability to vary blood flow to match metabolic activity. An extensive arborization of perivascular nerves also serves to modulate cerebral blood flow, so-called neurogenic regulation. Central to these three paradigms are two cell types: endothelium and astrocytes. The endothelium produces several vasoactive factors that are germane to the regulation of cerebral blood flow: nitric oxide, endothelium-dependent hyperpolarization factor, the eicosanoids, and the endothelins. Astrocytic foot processes directly abut the blood vessels, and play a key role in regulation of cerebral blood flow. Lastly, new research has been investigating cell-cell communication at the microvascular level. Several lines of evidence point to the ability of the larger proximal vessels to coordinate vasomotor responses downstream.

## 1. Introduction

Regulation of blood flow in the human brain is exceedingly complex. There exist multiple overlapping regulatory paradigms and key structural components. The interaction of these components, as well as the components themselves, are not fully understood. Nonetheless, a great deal of progress has been made in this important field.

This paper will discuss the three main regulatory paradigms involved in the regulation of cerebral blood flow: cerebral autoregulation, flow-metabolism coupling, and neurogenic regulation. In addition, there are two cell types that have repeatedly been shown to play a central role in the regulation of cerebral blood flow: endothelial cells and astrocytes. Lastly, the role of microvascular communication is discussed.

## 2. Cerebral Pressure Autoregulation

**Figure 1:**
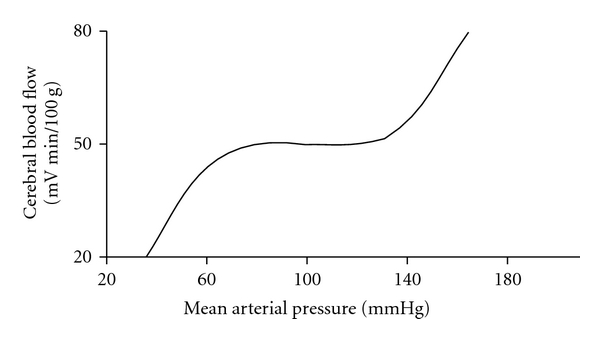
Idealized curve demonstrating cerebral pressure autoregulation.

The process whereby the cerebral arteries (specifically arterioles) maintain a constant blood flow (CBF) in the face of changing cerebral perfusion pressure (CPP) is referred to as *cerebral pressure autoregulation*. As shown below in Figure 1, between CPP pressures of 50–150 mm Hg CBF is relatively constant; above and below these values, however, CBF varies markedly with CPP. 

We view this phenomenon as independent of metabolic factors, thus this section is limited to changes in vascular tone as a result of CPP only. *Flow-metabolism* coupling is a distinct phenomenon that will be discussed in the following section.

The exact mechanism underlying cerebral pressure autoregulation continues to elude us. Several theories have been advanced, focusing on the endothelium, nerves, and the vascular smooth muscle itself. Because of the extensive investigation into the perivascular nerve fibers, discussion of this is relegated to a separate section below.

The endothelium is a dynamic source of a plethora of vasomodulatory molecules. In addition, it has been proposed that the endothelium has mechanoreceptor properties that allow it to contribute to cerebral autoregulation. The two main mechanical mechanisms that have been evaluated are shear stress and transmural pressure. Increased flow-velocity (shear stress) has been shown to induce vasoconstriction independent of transmural pressure [1]. This response is attenuated in blood vessels denuded of endothelium. A similar endothelium-dependent response to increases in transmural pressure has also been demonstrated. Harder reproduced this work and also found that arterial constriction was associated with smooth muscle depolarization [2]. Lastly, Rubanyi showed that perfusate isolated from arteries that had been subjected to increased transmural pressure was capable of inducing vasoconstriction in naïve vessels, implying some endothelial-derived factor [3].

Stretch responses have also been theorized to originate in smooth muscle cells. Originally formulated by Bayliss in 1902 [4], the so-called “myogenic hypothesis” of cerebral autoregulation focuses on the mechanoreceptor properties of smooth muscle cells themselves. The development in 1981 of isolated vessel techniques allowed the mechanisms to be separated from flow, neural, metabolic, and endothelial influences [5, 6]. Recent work has focused on the transduction mechanisms between myogenic stretch and subsequent vasoconstriction, particularly the role of stretch-activated ion channels. Since the first recordings of mechanosensitive ion channels in 1988 [7], a number of investigators have found evidence for these channels in vascular smooth muscle in a variety of tissues. The electrical properties of these channels strongly suggest that they are nonselective cation channels [8, 9]. The resultant membrane depolarization results in influx of Ca^++^ through voltage-gated Ca^++^ channels and smooth muscle constriction, a response that is abolished in the presence of inhibitors to voltage-gated Ca^++^ channels [10]. It has also recently been shown that not only the RhoA-Rho Kinase pathway plays a pivotal role in cerebral artery mechanotransduction, but also the pathway is more active at progressively higher levels of stretch [11]. Gokina and colleagues evaluated the effect of Rho kinase inhibition on pressure autoregulation in cerebral arteries in the rat. They found that administration of a specific inhibitor of Rho kinase (Y-27632) selectively inhibited pressure-induced rise in intracellular Ca^+^ as well as the development of myogenic tone. Studies have even demonstrated that calcium-independent mechanisms, so-called calcium sensitization, may be present as well. This occurs when agonists lead to muscle contraction without a corresponding rise in intracellular calcium [12]. 

## 3. Flow-Metabolism Coupling

For over a century it has been appreciated that cerebral blood flow varies with cerebral metabolism [13]. This has most recently been shown with several functional imaging modalities, such as PET scanning and BOLD fMRI [14]. So-called *flow-metabolism coupling* or *functional hyperemia* is perhaps the most clinically relevant of the CBF regulation paradigms, as cerebral tissue is among the least tolerant of ischemia. Interestingly enough, it has been shown that the generation of action potentials alone is not necessarily the main stimulus in flow-metabolism coupling; rather the interneuron milieu is capable of generating a significant stimulus even if the endpoint is a *decrease* in the frequency of action potentials [15, 16].

There are several molecules that investigators have focused on as possible links between neuronal activity and the regulation of cerebral blood flow. All are known to increase with synaptic transmission, either because they are involved in the process itself (in the case of K+ and H+) or because they are known metabolites (adenosine).

Potassium and hydrogen ions are produced by synaptic transmission, and it has been shown that elevation of these ions stimulates vasodilation [17, 18], thus providing a possible mechanism for neurovascular coupling. Potassium channels have been found in vascular smooth muscle and have been implicated in the effect of both K^+^ and H^+^ elevations [19]. In addition, ATP-sensitive K^+^-channels have been found in vascular smooth muscle, suggesting a direct link between neuronal activity and cerebral blood flow [20]. The well-known phenomenon of CO_2_ reactivity is mediated through the action of H^+^ on cerebral arteries, rather than CO_2_ itself. The transduction mechanism of H^+^ into vasodilation remains elusive, although nitric oxide (NO) has been implicated as the vasodilatory response to hypercapnia and acidosis is attenuated by NO inhibitors [21, 22]. 

Metabolites are an attractive option as messengers for flow-metabolism coupling, for obvious reasons. Because hypoxia and hypoglycemia do not affect activity-induced vasodilatations [23], attention has focused on other by-products of neuronal activity, most notably adenosine. Extracellular levels of adenosine rise sharply with neuronal activity, and topical application of adenosine to cerebral microcirculation causes vasodilation [24]. Furthermore, adenosine has been shown to be released in response to administration of glutamate, a major neurotransmitter in cerebral cortex [25]. Iliff et al. [26] showed that selective blockade of adenosine receptor (specifically receptor 2A) attenuated the vasodilatory response to glutamate administration. Furthermore, this blockade had no effect on either resting vessel diameter or on CO_2_ reactivity. Thus adenosine appears to play a significant role in glutamate-induced dilation of pial arterioles.

A significant body of evidence supports the function of NO in flow-metabolism coupling [24, 27, 28], although the exact pathway is not completely clear. A recent study by Lindauer et al. demonstrated that the vasodilatory response to whisker deflection was blocked by administration of NO inhibitors. However, the effect was restored by administration of cyclic guanosine monophosphate (cGMP) or NO donors, suggesting that NO itself is not the relevant mediator [29]. It has been theorized that NO acts as a permissive agent, supplying a basal amount of cGMP for other mediators (like adenosine) to utilize as second messengers [26].

## 4. Neurogenic Regulation of Cerebral Blood Flow (Box 1 in Figure 2)

A wealth of functional and histological evidence supports the existence of an extensive arborization of perivascular nerves that play a role in regulation of cerebral blood flow. The functional unit of endothelial cells, perivascular nerves, and astrocytes has been increasingly recognized as a complex network that can be considered as a single entity rather than separate subunits. Referred to as the *neurovascular unit*, this characterization has led to investigation into supporting the unit as a whole, rather than simply focusing on one aspect [30]. These nerves have diverse origins and neurotransmitters and can be broadly categorized into two categories: extrinsic and intrinsic.

Extrinsic perivascular innervation refers to vessel innervation outside of the brain parenchyma. Three main sources of extrinsic perivascular innervation have been identified: the trigeminal ganglion, the superior cervical ganglion, and the sphenopalatine ganglion. These ganglions carry sensory, sympathetic, and parasympathetic nerves, respectively. It has been hypothesized that the main role of the sympathetic nervous system is to offer an increased tone to maintain blood pressure below the upper limit of the autoregulatory mechanism [31, 32]. Thus pressures that would normally overwhelm autoregulation are well tolerated. The parasympathetic system is felt to play a role primarily in pathological states. Because of the central role the trigeminovascular system plays in pain sensation, it became an early focus for migraine. It was later discovered that calcitonin gene-related peptide (CGRP), a potent vasodilator, is released from trigeminal nerves [33]. It was thus theorized that the trigeminovascular system plays a role in counteracting vasoconstrictive influences. In addition, the triptan class of migraine abortive agents act presynaptically to prevent CGRP release, thereby preventing vascular engorgement and associated headache.

**Figure 2:**
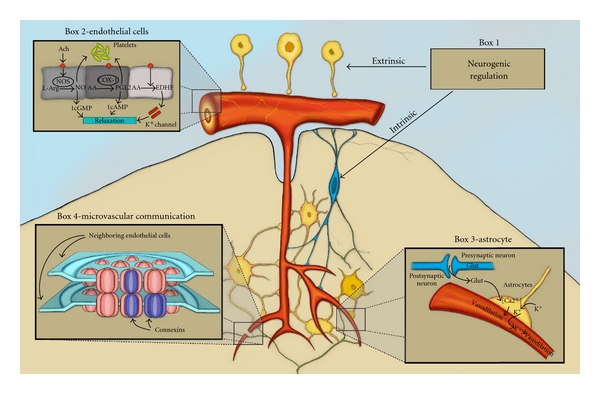
Comprehensive diagram demonstrating the multiple mechanisms of cerebrovascular control. Modified from Hamel et al., J AP with permission.

As shown in Figure 2, once the blood vessel dives deep into the parenchyma and leaves the virchow-robin space, they lose their extrinsic innervation and the intrinsic innervation begins. A new set of nerves takes over, arising both from distant pathways [34–36] and local interneurons [37]. The majority of these nerves do not abut directly on blood microvessels themselves; rather they connect to astrocyte foot processes. Depending on the area stimulated, an increase or decrease in CBF can be elicited. The nucleus basalis, locus coeruleus, and raphe nucleus have all been implicated as a source for innervation of cerebral microvasculature. At present, it is not entirely clear if these neurons directly contact the microvessels or if the signal is transduced through astrocytic foot-processes [38]. 

In addition to distant subcortical pathways, local interneurons also play a role in the regulation of microvascular tone. It has recently been shown that stimulation of GABAnergic interneurons causes vasodilation of regional microvessels [39]. Furthermore, interneurons also seem to be necessary to transduce the signal from distant subcortical regions described above. This suggests a mechanism for feed-forward vasodilation [40].

## 5. Endothelium (Box 2 in Figure 2)

The cerebrovascular endothelium plays a central role in the regulation of cerebral blood flow. Once thought to simply be an inert antithrombotic barrier, the endothelium is now appreciated as a dynamic organ that acts as a physiologic bridge between the blood vessel lumen and the surrounding smooth muscle. At present, this bridge is thought of as comprising 4 main chemical systems: nitric oxide (NO), endothelium-derived hyperpolarization factor (EDHF), the eicosanoids, and the endothelins. 

Much of the interest in the endothelium began with the discovery of nitric oxide (NO) as the endothelium-derived relaxing factor by Furchgott [41] and Ignarro [42] in 1988. NO is a diffusible second messenger that activates guanylate cyclase (GC), present in smooth muscle cells. Guanylate cyclast in turn synthesizes cGMP which causes smooth muscle relaxation through protein kinase G (PKG) activation of K^+^ channels and/or closure of voltage-gated calcium channels. 

The enzyme that produces NO, nitric oxide synthase (NOS) has several isoforms. Endothelial NOS (eNOS) is the isoform found in the cerebral blood vessels, specifically the endothelium [43–45]. Neuronal NOS (nNOS) is the isoform found in neurons. A third isoform, inducible NOS (iNOS) has been found in the brain under pathological situations such as hypertension or exposure to endotoxin [46]. It is not thought to be active in the brain under normal conditions. Immunohistochemistry has localized eNOS to the endothelium, yet administration of a NOS inhibitor (L-NMMA) to denuded arteries produces vasoconstriction [47, 48]. This provides evidence for the physiological significance of nNOS, although the relative contribution of eNOS versus nNOS to resting tone of the cerebral vasculature is not known. 

Despite the dominance of the NO paradigm in endothelial-dependent vasodilatation, it appears that there is a second mechanism that also operates in the endothelium to cause vasodilatation. This is based on observations that when NO- and eicosanoid-based pathways are fully inhibited, further dilatory capacity remains [49–51]. Similar to the story of EDRF prior to the discovery of NO, so-called EDHF represents an as of yet unelucidated pathway that may be another diffusible molecule (the present terminology of “factor” is misleading based on the present data). This pathway is characterized by hyperpolarization of the vascular smooth muscle and is inhibited by K^+^-channel blockers. Golding et al. defined EDHF as a dilation pathway that [1] requires endothelium, [2] is distinct from eicosanoid or NO pathways, [3] dilates via hyperpolarization of vascular smooth muscle, and [4] involves K^+^-channel activation [52]. It is known that the EDHF pathway begins with an *increase* in endothelial Ca^+2^ stores and ends with a *decrease* in smooth muscle Ca^+2^ stores. How these two events are linked is a matter of continuing study. At present, there are 4 main possibilities: eicosanoids, K^+^, gap junctions, and hydrogen peroxide [53]. 

The eicosanoids are a group of vasoactive chemical mediators that are derived from arachidonic acid. Three main enzyme systems have been identified: cyclooxygenase (COX), lipoxygenase (LOX), and epoxygenase (EPOX). Arachidonic acid is formed from membrane phospholipids by lipases and is used as a substrate for the aforementioned enzyme systems [54]. 

It should be emphasized that these enzyme systems are not limited to the endothelium; rather they are active in a variety of tissues, notably platelets. Furthermore, some products cause vasoconstriction and others cause vasodilatation. The differential concentrations of the enzymes and their isoforms in different locations determines the overall effect on cerebral blood flow. Although all three enzyme systems have been investigated extensively in the systemic vasculature, COX is the best understood in the cerebral vasculature—at present, the role of the LOX and EPOX systems in the cerebral vasculature is poorly understood. Three COX isoforms have been found to exist in the cerebral vasculature [55–58]. Of the multiple metabolites produced by COX, the vasodilators prostacyclin (PGI_2_) and prostaglandin E_2_ (PGE_2_) are predominant in normal endothelium [59]. While important in pathologic conditions, it appears that under normal physiology the COX system is less dominant, with the NO and EDHF systems described above being the predominant vasodilatation paradigms [60]. The vasoconstrictive COX metabolites are presently thought to be most relevant in pathological situations such as traumatic brain injury and subarachnoid hemorrhage.

The fourth major chemical system active in the endothelium is that of the endothelins. This system is comprised of two receptors (ET_A_ and ET_B_) and three ligands (ET-1, ET-2, ET-3). The effect of the ligands seems to depend on the receptor rather than the ligand. ET_A_ receptors are found predominantly in vascular smooth muscle, are stimulated by ET-1 and ET-2, and mediate vasoconstriction. ET_B_ receptors are found predominantly within the endothelium, are stimulated by all three ligands, and mediate vasodilatation [61, 62]. Of the three ligands, ET-1 appears to play the biggest role in the regulation of cerebral blood flow. It can either be produced via cleavage of proendothelin, or “big ET-1”, or produced de novo from mRNA in cerebrovascular endothelial cells [63]. In the presence of an intact endothelium, ET-1 binds to ET_B_ and causes vasodilation [64]; in vessels denuded of endothelium, ET-1 binds to ET_A_ and causes vascoconstriction [65, 66]. The picture is somewhat complicated by the fact that ET_A_ receptors may be found in the endothelium [67] and ET_B_ receptors may be found in smooth muscle [68, 69]. The vasodilatory action of ET_B_ receptors is mediated by NO [70]. Interestingly, there is evidence of a balancing paradigm between the constricting actions of ET-1 and NO-mediated vasodilation. ET-1 secretion has been shown to be stimulated by several molecules known to be connected with NO-induced vasodilation. However, topical administration of ET receptor blockers does not result in an increase in CBF. Furthermore, the long lasting effects of the endothelins make them poorly suited for the minute to minute regulation of CBF. It is presently thought that endothelins do not contribute in a major way to resting CBF under normal physiologic conditions, although they have been shown to play a major role in several pathological conditions, most notably cerebral ischemia and cerebral vasospasm [71]. 

## 6. Astrocytes (Box 3 in Figure 2)

Astrocytes are in a unique anatomical position to effect CBF; their processes extensively ensheath brain capillaries [38], thereby physically linking the cerebral microvasculature with synapses. Their primary role was originally thought to involve extracellular K^+^ buffering. More recently, in vitro studies have shown astrocytes to be capable of cell-to-cell communication through gap junctions, suggesting a possible role for modulation of neuronal and vascular function. 

Early work on the mechanism of astrocytic involvement of cerebral blood flow regulation focused on potassium. Astrocytes were known to uptake excess extracellular potassium, and Newman et al. showed that the ions were then shunted to the endfeet processes [72]. Given the location of astrocytic foot processes on the cerebral vasculature and the vasodilatory effects of K^+^, it was theorized that this was a mechanism whereby neuronal activity was linked to cerebral vasodilation. Subsequent work by Paulson showed that this theory fits the temporal relationship of K^+^ increases to vasodilation better than alternative theories involving simple potassium diffusion [18].

Work by Zonta et al. using brain slice preparations found that electrical stimulation of astrocytes resulted in an increase in intracellular Ca^++^ followed by vasodilation of arterioles contacted by foot processes of that astrocyte [73]. This response was mimicked by administration of glutamate agonists and attenuated by administration of glutamate antagonists. Astrocytes are known to express a subtype of glutamate receptor that results in an increase in intracellular Ca^++^, thus it was inferred that astrocytic foot processes sensed the synaptic activity and then responded to that activity by inducing vasodilation of the appropriate blood vessels [26].

## 7. Microvascular Communication (Box 4 in Figure 2)

Much current interest is focused on the role microvascular bed in the regulation of cerebral blood flow. Specifically, there is evidence that communication within the blood vessels themselves at the microvascular level plays a key role in the overall regulation of blood flow in the brain. Since there is a substantial increase in resistance at the arteriole level, the proximal vessels must coordinate with the microvasculature in order to ensure adequate microperfusion. Several lines of evidence have been presented proving that this occurs.

Rosenblum et al. first presented in vivo evidence for the presence of coordinated vasomotor responses (CVR) in mouse pial arterioles [74], by demonstrating that the local constriction response initiated by micropipette application of uridine triphosphate could travel for 300 *μ*m or more upstream from the point of application. Moreover, conduction could be interrupted by local vascular wall injury (light-dye technique). Subsequently, Dietrich et al. [75] described conducted vasodilation in penetrating arterioles isolated from the rat brain to various vasoactive agents, including adenosine and ATP. They also suggested that endothelial cells appeared to play a key role in the conducted responses of intracerebral arterioles to potassium [76]. The same group [77] also found attenuation of conducted vasodilation by oxyhemoglobin treatment, suggesting a role by vascular conduction in the ischemic conditions developed after subarachnoid hemorrhage.

Pioneering studies by Segal and Duling [78, 79] sparked interest in CVR as a mechanism for coordinating vasomotor responses. A simple experimental paradigm, used by these investigators to investigate vascular conduction in peripheral vessels, is to follow the longitudinal spread of vasomotor responses in arterioles to discrete stimulation of arterioles. Thus, restricted application of vasoactive substances to arteriolar segments induces both direct effects and secondary conducted vasomotor responses [78, 79]. The conducted dilation response could not be explained by simple diffusion or by neural innervation and is independent of blood flow and pressure, because occlusion of an arteriole to create a sealed sac did not affect the propagated dilation. Thus, the pathway of signal conduction is located exclusively in vessel wall components (endothelium and/or smooth muscle).

Cx40 and Cx43 have been identified in smooth muscle and endothelium, whereas Cx45 has only been reported in smooth muscle [80, 81]. Because connexins have electrical and conductive properties that are different from one another [81, 82], selective upregulation and downregulation of different connexins, that is, the change in expression profile, likely shapes the change in vasomotor conductivity after an ischemic insult. 

Initial studies showed that the longitudinal spread of arteriolar response to focal agonist application resembled electrical decay along a cable [79], suggesting that electrical signals travel by passive spread of current between cells in the vessel wall. This electrotonic current travel therefore should vary with transmembrane and axial resistances. However, in vivo observations of conducted vasodilation indicate that some vasomotor responses exhibit little decay [83, 84]. For example, hyperpolarization induced by acetylcholine in arteriolar networks of the hamster cheek pouch in vivo was maintained, and even grew, as it traveled [84]. The mechanism for generating the additional current to aid the spread of hyperpolarization may involve inward rectifier K^+^channels [85, 86] and/or Na^+^/K^+^  ATPase in smooth muscle cells [87]. In cerebral penetrating arterioles, barium chloride (BaCl_2_) attenuated both conducted dilation and constriction responses to K^+^, suggesting that K_ir_ may be involved in CVR in the cerebrovasculature as well [88]. 

## 8. Conclusion

The regulation of blood flow in the brain is exceedingly complex and only beginning to be elucidated. We have attempted to broadly outline the major categories of mechanisms discovered so far, focusing on pressure autoregulation, metabolic regulation, and neurogenic regulation. Central to all three groups is the neurovascular unit, composed of endothelial cells, neurons, and astrocytes. The role of this substrate in a variety of pathologic states characterized in part by failure of cerebral blood flow control (stroke, traumatic brain injury, hypertension, Alzheimer's disease) is the target of intense investigation and underscores the need to explore the control of blood flow at level of the microcirculation as well.

## References

[1] Edvinsson L, MacKenzie E, McCulloch J (1993). *Cerebral Blood Flow and Metabolism*.

[2] Harder DR (1987). Pressure-induced myogenic activation of cat cerebral arteries is dependent on intact endothelium. *Circulation Research*.

[3] Rubanyi GM, Freay AD, Kauser K, Johns A, Harder DR (1990). Mechanoreception by the endothelium: mediators and mechanisms of pressure- and flow-induced vascular responses. *Blood Vessels*.

[4] Bayliss WM (1902). On the local reactions of the arterial wall to changes of internal pressure. *Journal of Physiology*.

[5] Jackson PA, Duling BR (1989). Myogenic response and wall mechanics of arterioles. *American Journal of Physiology*.

[6] Kuo L, Davis MJ, Chilian WM (1988). Myogenic activity in isolated subepicardial and subendocardial coronary arterioles. *American Journal of Physiology*.

[7] Kirber MT, Walsh JV, Singer JJ (1988). Stretch-activated ion channels in smooth muscle: a mechanism for the initation of stretch-induced contraction. *Pflugers Archiv*.

[8] Davis MJ, Donovitz JA, Hood JD (1992). Stretch-activated single-channel and whole cell currents in vascular smooth muscle cells. *American Journal of Physiology*.

[9] Setoguchi M, Ohya Y, Abe I, Fujishima M (1997). Stretch-activated whole-cell currents in smooth muscle cells from mesenteric resistance artery of guinea-pig. *Journal of Physiology*.

[10] Davis MJ, Hill MA (1999). Signaling mechanisms underlying the vascular myogenic response. *Physiological Reviews*.

[11] Gokina NI, Park KM, McElroy-Yaggy K, Osol G (2005). Effects of Rho kinase inhibition on cerebral artery myogenic tone and reactivity. *Journal of Applied Physiology*.

[12] Johnson RP, El-yazbi AF, Takeya K, Walsh EJ, Walsh MP, Cole WC (2009). Ca2+ sensitization via phosphorylation of myosin phosphatase targeting subunit at threonine-855 by Rho kinase contributes to the arterial myogenic response. *Journal of Physiology*.

[13] Roy CS, Sherrington CS (1890). On the regulation of the blood-supply of the brain. *Journal of Physiology*.

[14] Villringer A, Dirnagl U (1995). Coupling of brain activity and cerebral blood flow: basis of functional neuroimaging. *Cerebrovascular and Brain Metabolism Reviews*.

[15] Logothetis NK, Pauls J, Augath M, Trinath T, Oeltermann A (2001). Neurophysiological investigation of the basis of the fMRI signal. *Nature*.

[16] Mathiesen C, Caesar K, Akgören N, Lauritzen M (1998). Modification of activity-dependent increases of cerebral blood flow by excitatory synaptic activity and spikes in rat cerebellar cortex. *Journal of Physiology*.

[17] Nielsen AN, Lauritzen M (2001). Coupling and uncoupling of activity-dependent increases of neuronal activity and blood flow in rat somatosensory cortex. *Journal of Physiology*.

[18] Paulson OB, Newman EA (1987). Does the release of potassium from astrocyte endfeet regulate cerebral blood flow?. *Science*.

[19] Buerk DG, Ances BM, Greenberg JH, Detre JA (2003). Temporal dynamics of brain tissue nitric oxide during functional forepaw stimulation in rats. *Neuroimage*.

[20] Quayle JM, Nelson MT, Standen NB (1997). ATP-sensitive and inwardly rectifying potassium channels in smooth muscle. *Physiological Reviews*.

[21] Iadecola C (1992). Does nitric oxide mediate the increases in cerebral blood flow elicited by hypercapnia?. *Proceedings of the National Academy of Sciences of the United States of America*.

[22] Persson PB (1996). Modulation of cardiovascular control mechanisms and their interaction. *Physiological Reviews*.

[23] Imai Y, Ohkubo T, Tsuji I, Satoh H, Hisamichi S (1999). Clinical significance of nocturnal blood pressure monitoring. *Clinical and Experimental Hypertension*.

[24] Pelligrino DA, Gay RL, Baughman VL, Wang Q (1996). NO synthase inhibition modulates NMDA-induced changes in cerebral blood flow and EEG activity. *American Journal of Physiology*.

[25] Hoehn K, White TD (1990). Role of excitatory amino acid receptors in K+- and glutamate-evoked release of endogenous adenosine from rat cortical slices. *Journal of Neurochemistry*.

[26] Iliff JJ, D’Ambrosio R, Ngai AC, Winn HR (2003). Adenosine receptors mediate glutamate-evoked arteriolar dilation in the rat cerebral cortex. *American Journal of Physiology*.

[27] Faraci FM, Breese KR (1993). Nitric oxide mediates vasodilatation in response to activation of N- methyl-D-aspartate receptors in brain. *Circulation Research*.

[28] Meng W, Tobin JR, Busija DW (1995). Glutamate-induced cerebral vasodilation is mediated by nitric oxide through N-methyl-D-aspartate receptors. *Stroke*.

[29] Lindauer U, Megow D, Matsuda H, Dirnagl U (1999). Nitric oxide: a modulator, but not a mediator, of neurovascular coupling in rat somatosensory cortex. *American Journal of Physiology*.

[30] Lok J, Gupta P, Guo S (2007). Cell-cell signaling in the neurovascular unit. *Neurochemical Research*.

[31] Chillon JM, Baumbach GL (2002). Autoregulation: arterial and intracranila pressure. *Cerebral Blood Flow and Metabolism*.

[32] Goadsby PJ, Edvinsson L (2002). *Neurovascular control of the cerebral circulation*.

[33] Waeber C, Moskowitz MA (2005). Migraine as an inflammatory disorder. *Neurology*.

[34] Cohen Z, Bonvento G, Lacombe P, Hamel E (1996). Serotonin in the regulation of brain microcirculation. *Progress in Neurobiology*.

[35] Hamel E (2004). Cholinergic modulation of the cortical microvascular bed. *Progress in Brain Research*.

[36] Iadecola C (2004). Neurovascular regulation in the normal brain and in Alzheimer’s disease. *Nature Reviews Neuroscience*.

[37] Vaucher E, Tong XK, Cholet N, Lantin S, Hamel E (2000). GABA neurons provide a rich input to microvessels but not nitric oxide neurons in the rat cerebral cortex: A means for direct regulation of local cerebral blood flow. *Journal of Comparative Neurology*.

[38] Simard M, Arcuino G, Takano T, Liu QS, Nedergaard M (2003). Signaling at the gliovascular interface. *Journal of Neuroscience*.

[39] Cauli B, Tong XK, Rancillac A (2004). Cortical GABA interneurons in neurovascular coupling: Relays for subcortical vasoactive pathways. *Journal of Neuroscience*.

[40] Iadecola C, Arneric SP, Baker HD, Tucker LW, Reis DJ (1987). Role of local neurons in cerebrocortical vasodilation elicited from cerebellum. *American Journal of Physiology*.

[41] Furchgott R (1988). *Studies on Relaxation of Rabbit Aorta by Sodium Nitrite: The Basis for the Proposal that the Acid-Activatable Inhibitory Factor from Retractor Penis is Organic Nitrite and the Endothelium-Derived Relaxing Factor is Nitric Oxide*.

[42] Ignarro LJ, Buga GM, Wood KS, Byrns RE, Chaudhuri G (1987). Endothelium-derived relaxing factor produced and released from artery and vein is nitric oxide. *Proceedings of the National Academy of Sciences of the United States of America*.

[43] Benyó Z, Lacza Z, Hortobágyi T, Görlach C, Wahl M (2000). Functional importance of neuronal nitric oxide synthase in the endothelium of rat basilar arteries. *Brain Research*.

[44] Brody C (1999). Nurses can lead the charge for safer i.v. bags. *The American nurse*.

[45] Fleming I, Busse R (1999). NO: the primary EDRF. *Journal of Molecular and Cellular Cardiology*.

[46] Hernanz R, Briones AM, Alonso MJ, Vila E, Salaices M (2004). Hypertension alters role of iNOS, COX-2, and oxidative stress in bradykinin relaxation impairment after LPS in rat cerebral arteries. *American Journal of Physiology*.

[47] Faraci FM, Brian JE (1994). Nitric oxide and the cerebral circulation. *Stroke*.

[48] Katusic ZS (1991). Endothelium-independent contractions to N(G)-monomethyl-L-arginine in canine basilar artery. *Stroke*.

[49] Hutcheson IR, Chaytor AT, Evans WH, Griffith TM (1999). Nitric oxide-independent relaxations to acetylcholine and A23187 involve different routes of heterocellular communication: role of gap junctions and phospholipase A2. *Circulation Research*.

[50] You J, Johnson TD, Marrelli SP, Bryan RM (1999). Functional heterogeneity of endothelial P2 purinoceptors in the cerebrovascular tree of the rat. *American Journal of Physiology*.

[51] You J, Johnson TD, Marrelli SP, Mombouli JV, Bryan RM (1999). P(2u) receptor-mediated release of endothelium-derived relaxing factor/nitric oxide and endothelium-derived hyperpolarizing factor from cerebrovascular endothelium in rats. *Stroke*.

[52] Golding EM, Marrelli SP, You J, Bryan RM (2002). Endothelium-derived hyperpolarizing factor in the brain: a new regulator of cerebral blood flow?. *Stroke*.

[53] Bryan RM, You J, Golding EM, Marrelli SP (2005). Endothelium-derived hyperpolarizing factor: a cousin to nitric oxide and prostacyclin. *Anesthesiology*.

[54] Bogatcheva NV, Sergeeva MG, Dudek SM, Verin AD (2005). Arachidonic acid cascade in endothelial pathobiology. *Microvascular Research*.

[55] Chandrasekharan NV, Dai H, Roos KLT (2002). COX-3, a cyclooxygenase-1 variant inhibited by acetaminophen and other analgesic/antipyretic drugs: cloning, structure, and expression. *Proceedings of the National Academy of Sciences of the United States of America*.

[56] Kis B, Snipes A, Bari F, Busija DW (2004). Regional distribution of cyclooxygenase-3 mRNA in the rat central nervous system. *Molecular Brain Research*.

[57] Kis B, Snipes JA, Isse T, Nagy K, Busija DW (2003). Putative cyclooxygenase-3 expression in rat brain cells. *Journal of Cerebral Blood Flow and Metabolism*.

[58] Kis B, Snipes JA, Simandle SA, Busija DW (2005). Acetaminophen-sensitive prostaglandin production in rat cerebral endothelial cells. *American Journal of Physiology*.

[59] Moore SA, Spector AA, Hart MN (1988). Eicosanoid metabolism in cerebromicrovascular endothelium. *American Journal of Physiology*.

[60] You J, Golding EM, Bryan RM (2005). Arachidonic acid metabolites, hydrogen peroxide, and EDHF in cerebral arteries. *American Journal of Physiology*.

[61] Brian JE, Faraci FM, Heistad DD (1996). Recent insights into the regulation of cerebral circulation. *Clinical and Experimental Pharmacology and Physiology*.

[62] Salom JB, Torregrosa G, Alborch E (1995). Endothelins and the cerebral circulation. *Cerebrovascular and Brain Metabolism Reviews*.

[63] Yoshimoto S, Ishizaki Y, Kurihara H (1990). Cerebral microvessel endothelium is producing endothelin. *Brain Research*.

[64] Kitazono T, Heistad DD, Faraci FM (1995). Dilatation of the basilar artery in response to selective activation of endothelin B receptors in vivo. *Journal of Pharmacology and Experimental Therapeutics*.

[65] Adner M, You J, Edvinsson L (1993). Characterization of endothelin-A receptors in the cerebral circulation. *NeuroReport*.

[66] Salom JB, Torregrosa G, Barbera MD, Jover T, Alborch E (1993). Endothelin receptors mediating contraction in goat cerebral arteries. *British Journal of Pharmacology*.

[67] Stanimirovic DB, Yamamoto T, Uematsu S, Spatz M (1994). Endothelin-1 receptor binding and cellular signal transduction in cultured human brain endothelial cells. *Journal of Neurochemistry*.

[68] Fukuroda T, Ozaki S, Ihara M, Ishikawa K, Yano M, Nishikibe M (1994). Synergistic inhibition by BQ-123 and BQ-788 of endothelin-1-induced contractions of the rabbit pulmonary artery. *British Journal of Pharmacology*.

[69] Sudjarwo SA, Hori M, Takai M, Urade Y, Okada T, Karaki H (1993). A novel subtype of endothelin B receptor mediating contraction in swine pulmonary vein. *Life Sciences*.

[70] Namiki A, Hirata Y, Ishikawa M, Moroi M, Aikawa J, Machii K (1992). Endothelin-1- and endothelin-3-induced vasorelaxation via common generation of endothelium-derived nitric oxide. *Life Sciences*.

[71] Vatter H, Konczalla J, Seifert V (2011). Endothelin related pathophysiology in cerebral vasospasm: what happens to the cerebral vessels?. *Acta Neurochirurgica Supplementum*.

[72] Newman EA, Frambach DA, Odette LL (1984). Control of extracellular potassium levels by retinal glial cell K+ siphoning. *Science*.

[73] Zonta M, Sebelin A, Gobbo S, Fellin T, Pozzan T, Carmignoto G (2003). Glutamate-mediated cytosolic calcium oscillations regulate a pulsatile prostaglandin release from cultured rat astrocytes. *Journal of Physiology*.

[74] Rosenblum WI, Weinbrecht P, Nelson GH (1990). Propagated constriction in mouse pial arterioles: Possible role of endothelium in transmitting the propagated response. *Microcirculation, Endothelium and Lymphatics*.

[75] Dietrich HH, Kajita Y, Dacey RG (1996). Local and conducted vasomotor responses in isolated rat cerebral arterioles. *American Journal of Physiology*.

[76] Horiuchi T, Dietrich HH, Hongo K, Dacey RG (2002). Mechanism of extracellular K+-induced local and conducted responses in cerebral penetrating arterioles. *Stroke*.

[77] Kajita Y, Dietrich HH, Dacey RG (1996). Effects of oxyhemoglobin on local and propagated vasodilatory responses induced by adenosine, adenosine diphosphate, and adenosine triphosphate in rat cerebral arterioles. *Journal of Neurosurgery*.

[78] Segal SS, Duling BR (1986). Communication between feed arteries and microvessels in hamster striated muscle: Segmental vascular responses are functionally coordinated. *Circulation Research*.

[79] Segal SS, Duling BR (1986). Flow control among microvessels coordinated by intercellular conduction. *Science*.

[80] de Wit C, Hoepfl B, Wölfle SE (2006). Endothelial mediators and communication through vascular gap junctions. *Biological Chemistry*.

[81] Li X, Simard JM (2002). Increase in Cx45 gap junction channels in cerebral smooth muscle cells from SHR. *Hypertension*.

[82] Saez JC, Berthoud VM, Branes MC, Martinez AD, Beyer EC (2003). Plasma membrane channels formed by connexins: their regulation and functions. *Physiological Reviews*.

[83] Emerson GG, Neild TO, Segal SS (2002). Conduction of hyperpolarization along hamster feed arteries: augmentation by acetylcholine. *American Journal of Physiology*.

[84] Crane GJ, Neild TO, Segal SS (2004). Contribution of active membrane processes to conducted hyperpolarization in arterioles of hamster cheeck pouch. *Microcirculation*.

[85] Rivers RJ, Hein TW, Zhang C, Kuo L (2001). Activation of barium-sensitive inward rectifier potassium channels mediates remote dilation of coronary arterioles. *Circulation*.

[86] Jantzi MC, Brett SE, Jackson WF, Corteling R, Vigmond EJ, Welsh DG (2006). Inward rectifying potassium channels facilitate cell-to-cell communication in hamster retractor muscle feed arteries. *American Journal of Physiology*.

[87] Weston AH, Richards GR, Burnham MP, Félétou M, Vanhoutte PM, Edwards G (2002). K+-induced hyperpolarization in rat mesenteric artery: identification, localization and role of Na+/K+-ATPases. *British Journal of Pharmacology*.

[88] Edwards G, Dora KA, Gardener MJ, Garland CJ, Weston AH (1998). K+ is an endothelium-derived hyperpolarizing factor in rat arteries. *Nature*.

